# Gone with the wind: how state power and industrial policy in the offshore wind power sector are blowing away the obstacles to East Asia’s green energy transition

**DOI:** 10.1007/s43253-022-00082-7

**Published:** 2022-07-06

**Authors:** John Mathews, Elizabeth Thurbon, Sung-Young Kim, Hao Tan

**Affiliations:** 1 Macquarie Business School, Macquarie University, Sydney, Australia; 2 School of Social Sciences, University of New South Wales, Sydney, Australia; 3 School of Social Sciences, Macquarie University, Sydney, Australia; 4 Newcastle Business School, The University of Newcastle, Sydney, Australia

**Keywords:** State power, Industrial policy, Offshore wind power (OWP) sector, Developmental environmentalism, North East Asia, Green energy transition

## Abstract

Offshore wind power (OWP) is emerging as the fastest growing sector in the global race towards renewables, and likely to emerge in just a few years as the largest segment in absolute terms. It has grown from accounting for just 1% of wind power capacity in 2010 to 10% by 2019 and is anticipated to reach 20% early in the 2020s. The OWP sector involves heavy engineering in the building of huge turbines, steel and concrete platforms, and extensive subsea cabling that resembles the shipbuilding industry more than mass production of consumables like solar cells. European firms were early developers of OWP but are now witnessing the rise of strong competitors from Northeast Asia (China, Japan, Korea, Taiwan) as well as potentially the USA. We use the framework of developmental environmentalism to argue that NEAsian developmental state traditions are being extended in the way that firms and governments from the region are promoting OWP. We frame an evolutionary political economy (EPE) argument that characterizes these NEAsian states as in their different ways utilizing OWP as a sustainable and scalable renewable energy source, particularly when linked to green hydrogen production, and are developing a new generation of industrial policies to break down resistance to the energy transition. We frame an argument for these NEAsian transitions as continuing the developmental tradition in what has been described as developmental environmentalism, with state agencies playing a continuing role in setting new directions — in this case towards OWP. We contrast this framework with that of the widely recognized multilevel perspective (MLP) with its emphasis on bottom-up processes. We highlight the role played by fossil fuel companies in finding a place for themselves in the green transition, as they diversify from oil and gas operations (e.g., floating oil platforms) to renewable energy operations (offshore wind), redeploying their resources and capabilities to do so.

## Introduction

The world has witnessed a demonstration of the power of industrial strategy in promoting the emergence of strong industries in Northeast Asia (NEAsia) for both solar PV and wind power. Now it is about to witness a comparable demonstration as the NEAsian countries tackle a third wave of renewables, in the form of offshore wind power (OWP). Here the full panoply of industrial strategies is on display — from the setting of targets, phased in over time, to the initial subsidizing that is phased out over time, to the framing of financial assistance and promotion of financing consortia such as bank consortia as practiced by Taiwan. There is clear attention paid to the dynamics of market evolution, allowing (in the cases of Taiwan and Korea) for phasing out of monopoly arrangements for power generation and retail (although not transmission and distribution). It is striking how these elements of industrial strategy are all within the norms allowed by the World Trade Organization (WTO) (as compared with the Local Content Requirement (LCR) rules as used effectively by China in the early stages of its onshore wind power development); these strategies seem to be put fully into practice only by states in the NEAsian economies. This is a puzzle that poses a challenge to International Political Economy (IPE).

In this paper we build on suggestions that the discipline of IPE should play a central role in examining and explaining the global green transition that is under way (Kuzemko et al 2019; Newell 2019; Pearse 2020; Bettin 2020). We address the question as to why it is the NEAsian countries that utilize the full panoply of industrial strategies in driving the clean and green energy transition. We advance a tentative answer to this conundrum in the role that state strategies in promoting OWP are playing in dismantling remaining obstacles to the green energy transition in NEAsia. Indeed, we demonstrate how OWP is already providing a critical niche enabling oil companies to diversify and move from fossil fuel production to renewable power generation, such as in moving from offshore oil drilling to offshore wind power generation, particularly utilizing floating platforms. We frame the transition as one involving Schumpeterian “creative destruction” where in this case the destruction of some aspects of the IPE of energy (e.g., power generation utilizing thermal coal) is accompanied by creative innovation by oil companies (e.g., offshore generation of wind power) as well as entrepreneurial innovation by newcomers, all acting under state guidance. Our argument turns on the role played by these fossil fuel giants seeking to enter the renewable energy space, with potentially full state support — thereby removing one of the last obstacles standing in the way of the global green transition.

In contrast with the widely recognized multilevel perspective (MLP) in providing a framework for analyzing the green transition, we utilize the framework of developmental environmentalism that emphasizes state agencies playing a developmental role in guiding and facilitating the green transition. The setting of clear business targets by the NEAsian nations is part of their standard approach to industrial strategy — now involving green goals that are also business and export goals. The targets carry with them clear commitments regarding the emergence of industrial capabilities and the building of associated industrial supply chains — a strategy which had been applied with great success in the period of building onshore wind power, particularly by China. The role of subsidies has been moderated as the OWP industry evolves. Clear subsidized targets were included in the Taiwanese and Korean cases and early Chinese case — but now subsidies through feed-in tariffs (FiTs) are being dismantled as countries move to conducting auctions in awarding OWP contracts, in order to drive down costs and force market participants to be internationally competitive. While China is in the process of phasing out its subsidized national FiTs, its more complex governance structure allows provincial governments to maintain some subsidies and investment allowances to promote OWP activities. We highlight these points of continuity and variation in the various NEAsian strategies in comparison with strategies pursued in Europe and the USA (Li 2022; DeCastro et al 2019) in our analysis below.

An announcement in January 2020 by the Chinese government provided the clearest indication yet that the national authorities expect the OWP industry to grow rapidly and achieve self-reliance, enabling it to withstand international competition without continuing to lean on subsidies. In this paper we trace out the NEAsian initiatives in building this third wave of OWP, seeking their commonalities and competitive aspects, with a view to understanding why it is that Japan, Korea, Taiwan, and China regard industrial strategy as something to spur the green shift and accelerate the clean energy transition.

The argument we develop is that state-led developmental processes can be found in NEAsia as states seek to green their economies. Insofar as these continuing developmental tendencies are now pitched at greening the economy, working with environmental goals and not against them, the expression “developmental environmentalism” has been coined to describe this continuing and important role played by the state in the NEAsian clean and green transition (Kim and Thurbon 2015; Thurbon et al 2021). Our aim in this paper is to demonstrate the central role played by state agencies in navigating the evolutionary political economy of the energy transition in NEAsia, as well as the role played by incumbent fossil fuel companies. We point to the significance of OWP as providing a renewable sector where fossil fuel giants can and will play a role as protagonists.

Over the past decade, Northeast Asian countries have seized the lead in the global clean energy shift thanks not so much to government efforts to reduce carbon emissions (the approach characteristic of the West), but to these states’ deployment of dedicated green industrial strategies. These green industrial strategies — aimed at rapidly scaling up the first and second generation of renewables (solar and onshore wind) — and at securing competitive advantage in the clean, green industries of the future — have so far effectively helped to expedite the “creative” aspect of the “creative-destruction” dynamic that is (as argued by Thurbon et al. (2021)) the defining feature of the clean energy transition.

We argue that East Asian governments (and East Asia’s fossil fuel incumbents) recognize an opportunity in OWP. East Asian governments are now deploying dedicated industrial strategies to rapidly build their own OWP industries — and to overcome the last remaining obstacles to a comprehensive clean energy transition. We argue that these green industrial strategies are an extension of the developmental strategies from the past.

In this paper, we examine how these green industrial strategies are being operationalized — and the extent to which these OWP strategies are likely to blow away one of the most significant remaining obstacles to the region’s comprehensive clean energy transition by overcoming residual problems and providing an alternative to “destruction” for fossil fuel incumbents by bringing them on board with the clean energy shift. In doing so, we provide further insight into the ways in which East Asian governments are seeking to expedite the clean energy transition by intervening to manage both its creative and destructive aspects.

## The OWP sector: market growth and industrial dynamics

The global offshore wind power (OWP) industry is growing rapidly; it promises to be the largest renewable energy industry in the world, after solar PV and onshore wind power which may be considered as first and second waves of the renewable revolution. OWP is already overtaking fossil-fuelled industries like coal and gas as cost plummet. It is an industry where the heavy industrial aspects are most evident — more like shipbuilding (with the giant floating platforms and huge wind turbines) than mass production of solar cells. It is also an industry where even the major oil companies are starting to play an important role, bringing their vast store of capabilities and their political influence to bear on the global green shift (for example, translating their expertise in floating oil and gas platforms to floating wind power platforms). The global offshore wind power industry is one where Europe currently holds the lead (with the UK and Denmark holding firm) but where China, Korea, Taiwan, and Japan are also playing a growing part, not as laggards but as leaders in market growth and innovation. In other words, the OWP sector is a fascinating crucible of international business rivalry in innovation, industrialization, and geo-political positioning, in driving the global green shift.

**Fig. 1:**
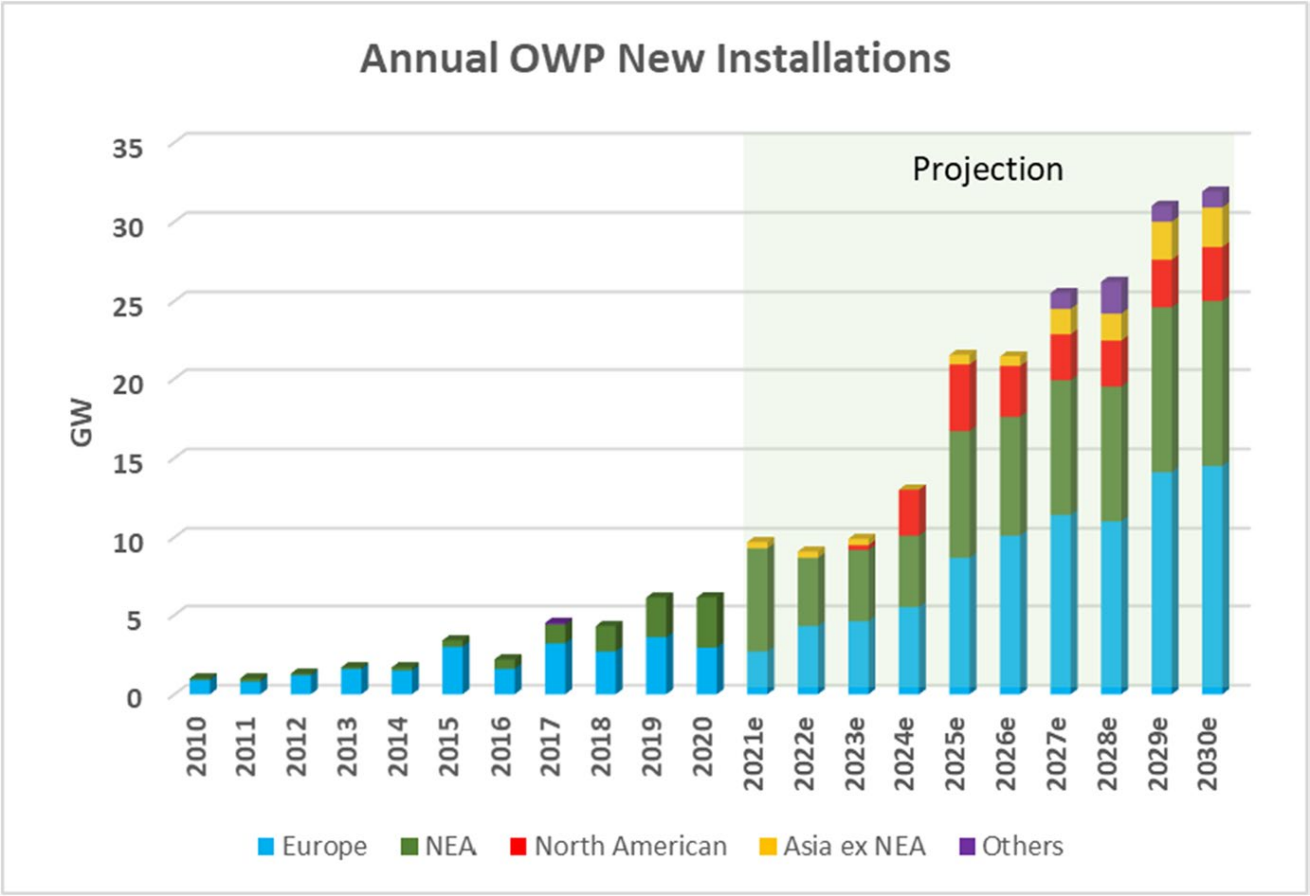
OWP annual new installations globally, 2010–2030(f). Source: Authors, based on GWEC data

According to the Global Wind Energy Council (GWEC), the year 2020 saw the OWP sector continuing its rapid growth, revealing it to be one of the world’s industries least affected by the COVID-19 pandemic. The year 2019 saw an historic record being set with the addition of 6.1 GW of offshore wind power capacity — 2.4 GW from China and 3.6 GW from Europe, marking these as the world leading regions driving the sector’s growth.^1^ The year 2020 saw a continuation of these trends, despite the Covid-19 pandemic, with a further 6.1-GW capacity added in OWP — with no less than 3.1 GW added from China, accounting for 50% of global capacity additions.^2^ Figure 1 shows how annual installations of offshore wind power (OWP) have grown, from around 1 GW per year a decade ago to more than 6 GW in 2019 and 2020. The Global Wind Energy Council (GWEC) sees annual installations growing to more than 30 GW within the next 10 years — an extremely rapid rate of growth. GWEC sees all the major centers of world economic activity involved in a competitive struggle — with European nations (and firms) maintaining their prominence all the way to 2025, and the NEAsian nations (China, Japan, Korea, Taiwan) growing rapidly over the course of the next decade, coming close to the European contribution, and contributions from North American competitors like GE also coming on strong. The GWEC projections also see some role for Asian involvement beyond that of NEAsia, encompassing countries such as Vietnam and India.

The OWP industry emerged just over a decade ago, reaching the important milestone of 1 GW in offshore installations by 2010. By 2020 new installations reached 6.1 GW, and the cumulative total reached 35.3 GW. From the early years, when OWP represented only 1% of the wind power sector, by 2019 OWP accounted for 10% of global installations. From 2015 to 2019 global market share of OWP rose from 5 to 10%, and is anticipated by GWEC to reach 20% by 2025. The industry is attracting widespread comment and appreciation.^3^

**Fig. 2:**
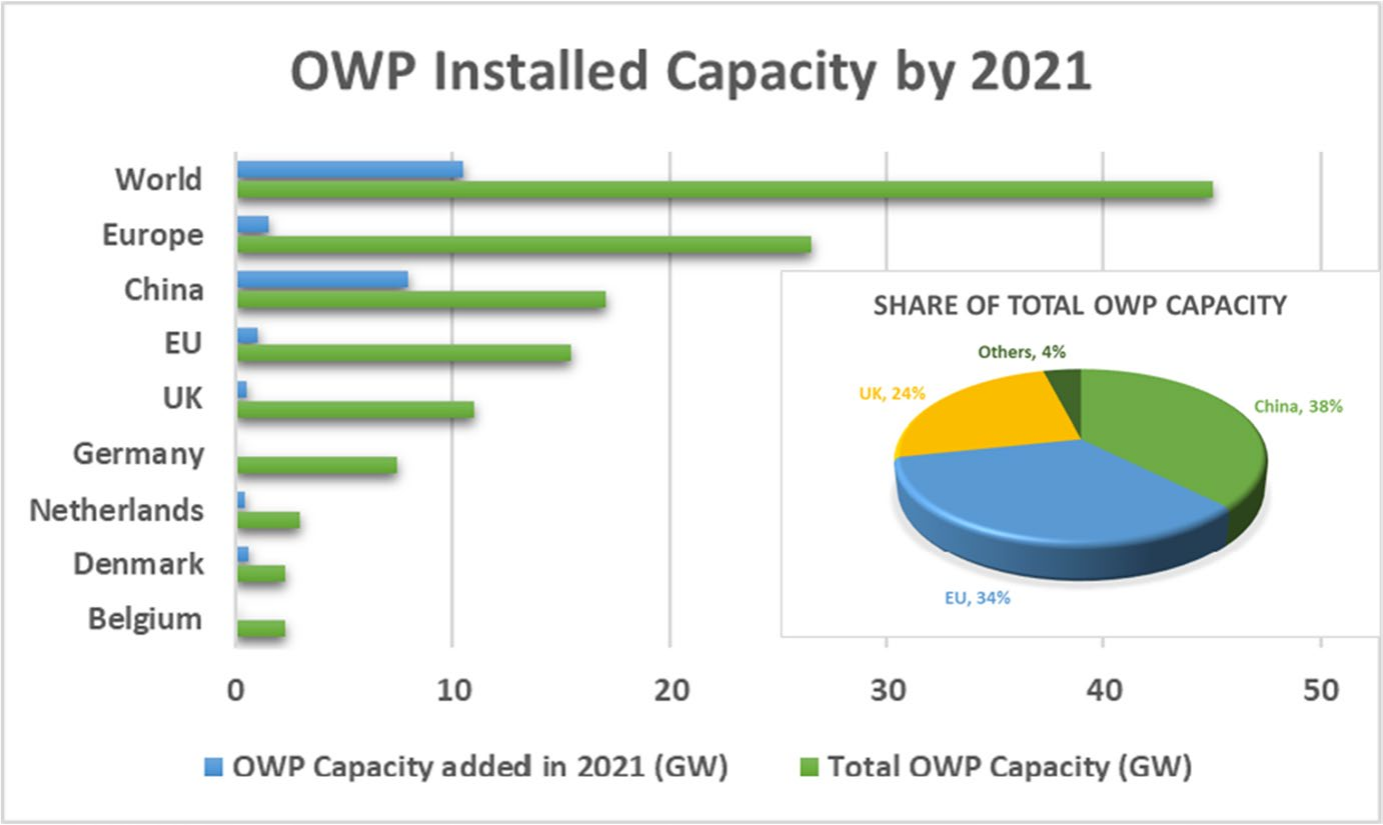
OWP installed capacity by 2021. Source: Authors, based on data from IEA

In its first years the industry was dominated by Europe, with firms like Vestas taking a leading role. China emerged as a player almost at the very beginnings of the industry, accounting for just 0.1 GW (100 MW) in 2010, and reaching 1 GW in newly installed capacity in 2017.^4^ Three NEAsian countries added capacity in 2019, marking them as significant players. China’s addition of 2.4 GW meant that it accounted for nearly 40% globally, followed by the UK (29%), Germany (18%), and Denmark (6%), while Taiwan and Japan emerged as serious players, accounting for 2%. The USA does not figure as a player at this stage. In 2020, China added 3.1 GW, a global share of 50.4%, followed by The Netherlands (24.6%), Belgium (11.6%), and the UK (8%). In terms of cumulative installations, of the 2020 total of 35.3 GW, the UK accounted for 28.9%, China for 28.3%, and Germany for 21.9%.^5^ Then in 2021 China took over as world leader in added new capacity; globally just over 10 GW was added, with China accounting for 8 GW of this. As shown in Fig. 2, the EU has now been overtaken by China in terms of cumulative installed capacity, with China accounting for 38% of cumulative capacity in 2021 and the EU for 34%.

Prospects for continued growth over the course of the next decade look sound. The GWEC sees the OWP Compound Annual Growth Rate (CAGR) maintaining 18.6% growth in annual installed capacity additions over the first half of the decade, moderating to 8.2% over the second half. Even so, this would mean that annual capacity additions would reach an estimated 32 GW by 2030. In the first half of the next decade the major share of growth is expected by GWEC to come from Europe as well as China and Taiwan.^6^ The top markets in the NEAsian region over the next decade (according to GWEC) are likely to be China (52 GW), Taiwan (10.5 GW), South Korea (7.9 GW), and Japan (7.4 GW) with Vietnam (5.2 GW) emerging as a player from SEAsia.

### Prospects over the next decade

Projections from the Global Wind Energy Council (GWEC) until 2030 are that there will be strong growth in the 2020s. China alone is listed as moving up from 4th to 2nd place globally, with a pipeline of OWP projects amounting to 26 GW by 2030. The USA and Brazil (a newcomer) occupy 3rd and 4th spots, with Taiwan holding to 5th spot, with a pipeline expanding from 9 to 15 GW.

Growth in China is anticipated to be prodigious. In October 2020, the *Wind Energy Beijing Declaration* was issued at the 2020 Beijing International Wind Energy Conference, suggesting that the government’s 14th five-year plan would include ambitious wind power (onshore and offshore) development targets, with cumulative capacity reaching 800 GW by 2030, and 3000 GW by 2060 (i.e., 3 TW within 40 years).^7^ China is far from being the only NEAsian country and government responding to the opportunities presented by OWP, and in this paper we point to the full panoply of targets and strategies that are being deployed by all four NEAsians in their pursuit of a strong position in the emerging OWP industry.

Central to the advance of OWP as a dominant renewable sector will be its links with hydrogen as a green energy carrier, with enormous prospects of its own. Many countries are pursuing hydrogen strategies with an emphasis on renewably powered electrolysis of water to produce “green hydrogen.” Supported by their governments, Japan and South Korea are creating markets for hydrogen and scaling up its production. In September 2020, Kobe/Kansai Hydrogen Utilization Council was formed by a number of Japanese firms, to develop CO_2_-free hydrogen utilization methods and establish a hydrogen supply chain in the Kobe/Kansai area.^8^ South Korean plans to develop three hydrogen-powered cities by 2022 and deploy hydrogen-based fuel cell vehicles (FCVs) on a wide scale.^9^ A German consortium announced in August 2020 that it would proceed with building of an electrolyzer that would run off OWP to produce green hydrogen via the *Westkuste100* project; this is a pointer to the probable centrality of OWP and renewables generally to the overall shift to a green hydrogen economy.^10^

## The floating platform (FP) OWP segment

Within the OWP sector there is a key niche occupied by floating platform OWP with its distinctive dynamics. Europeans are making headway at this frontier, but again we observe that China is rapidly situating itself as a player in this emerging and highly important sub-sector. The US National Renewable Energy Laboratory (NREL) projects that OWP could reach utility scale by 2024. There is already a pipeline of 24 GW of projects identified by NREL.^11^ Nearly 80% of the world’s offshore wind resources are available in waters that are deeper than 60 m. That is where floating offshore wind platforms come into their own.

The floating platform (FP) OWP sector is highly significant for two principal reasons. First, it is virtually without limits, as wind farm developers can build floating platforms off national coastlines and continental shelves at depths greater than 60 m, using well-known technologies such as tension leg platform (TLP), which allows them to generate wind power on virtually an open frontier. All the objections to wind power that apply to wind farms erected on land or near coastlines (unsightly, consuming too much space, killing birds) fall away once wind farms move farther away from the coast and towards the open sea. And second, the resort to floating platforms calls for capabilities and technological know-how already possessed by oil and gas companies. The emergence of the FP OWP sector opens up possibilities for oil and gas majors to diversify and enter the wind power sector, diversifying their business models from being oil and gas companies to becoming energy companies more generally. In this way, we see the FP segment of the emerging OWP industry as both sustainable and scalable, providing increasingly significant business advantages over other energy sources, particularly fossil fuel sources.

The oil and gas companies are frequently joining forces with specialist service providers to build the OWP farms, and particularly the floating platform variety. The French oil and gas giant **Total**, for example, announced in March 2020 that it had acquired an 80% stake in the 96-MW *Erebus* floating offshore wind power project to be constructed off the coat of Wales in the UK.^12^ Other oil majors have demonstrated similar commitments. The global oil major BP, for example, has entered into a JV with the Norwegian oil major Equinor to build FP OWP installations off the Brazilian coast.^13^

In NEAsia, **China** was an early starter with FP OWP initiatives, beginning with the Donghai Bridge project in 2010. Chinese floating platform OWP initiatives since then have been numerous. In 2020 the Chinese state-owned oil corporation CNOOC launched its first operating OWP platform, marking a decisive step toward diversification away from oil and gas towards renewable energy.^14^**Korea** too has been an active participant in floating platform OWP projects such as the 200-MW Donghae-1 floating platform OWP project being promoted by the Korean National Oil Co. (KNOC), using the Donghae-1 gas field production facilities.^15^**Japan** has emerged as a global leader in the development of floating offshore wind technology and aims to export its technology in the future.^16^ “Fukushima Forward”-floating offshore wind farm demonstrator, the experimental research project funded by the Ministry of Economy, Trade and Industry (METI), was finalized in July 2020 by successfully siting a 5-MW Hitachi wind turbine on a floating platform off the coast of Fukushima prefecture.^17^ The Japanese energy giant Mitsubishi Heavy Industries has joined forces with the European OWP leader Vestas to build offshore wind turbines in the USA and Asia, through the state-led joint venture MIHI-VOW.^18^ For its part, **Taiwan** has targeted the OWP sector as its next major industry, utilizing the full panoply of industrial strategy measures perfected in Taiwan’s earlier industrial development.

## Cost reductions associated with learning curves

**Fig. 3:**
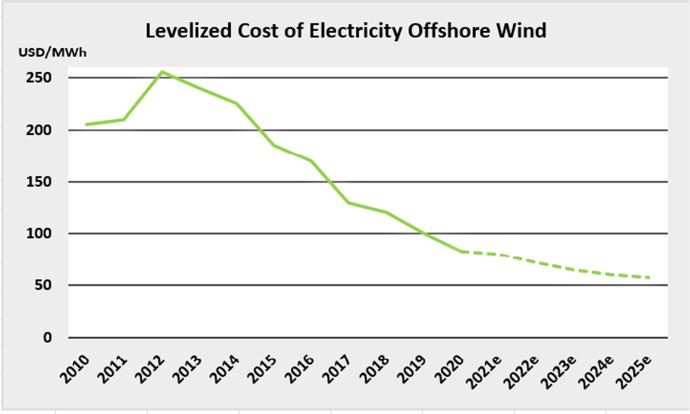
Levelized cost of electricity from offshore wind, 2010–2020. Source: Authors, based on GWEC data

Like other renewable sources of power that are associated with manufactured products, OWP operations are plunging in cost. Costs began at around $250 per MWh in 2012 and have fallen rapidly to $100 per MWh by 2019, continuing this downward trend into 2020 (see Fig. 3). This is a rate of cost reduction associated with a rapid learning curve (experience curve) that is characteristic of manufactured devices and their increasing returns; it is now demonstrated in the development of renewables, given the increasing instability of global fossil fuel supplies — a process referred to as “manufacturing renewables to build energy security” (Mathews and Tan 2014). In contrast, such learning curves are less apparent in mining and drilling operations which have diminishing returns.

Industry sources indicate that costs for generating OWP from fixed bottom installations have reduced from 17 to 18 c per kWh ($170–$180 per MWh) in 2015 to an anticipated 5–6 c per kWh ($50–$60 per MWh) in 2025 (see Fig. 3). Meanwhile costs for floating OWP are anticipated to be falling to $60 per MWh by 2030, with further reductions anticipated beyond that.^19^

If one accepts that renewable devices are subject to the same learning curves as other forms of manufacturing, it is possible to predict continuing cost reductions in the emergent OWP sector, reaching grid parity with electric power produced by burning fossil fuels early in the 2020s. It is striking how all the NEAsian proponents of the new OWP sector are counting on these anticipated cost reductions in devising their industrial strategies for the clean energy transition.

## NEAsian competitive performance: Taiwan, Korea, Japan, and China

All four NEAsian countries demonstrate market management skills in seeding and growing the OWP sector in their jurisdictions — setting up their anticipated international prominence over the course of the next decade. The striking feature of these NEAsian success stories is that the emergence of the OWP industry in each case is the result of clear and consistent industrial strategy, setting clear growth and export targets for the sector, rather than solely the carbon reduction targets that are commonly deployed in other jurisdictions. The NEAsian economies achieve advanced carbon reduction goals in any case — but they do so implicitly, while tackling explicit industrial targets in the first instance. All the stated targets depend (implicitly) on anticipated cost reductions, brought about by the learning curves associated with the manufacturing activities that drive the OWP sector.

### Taiwan

Taiwan exemplifies the NEAsian approach to industry building, as it methodically sets in place all the elements needed for a strong new sector like OWP — and setting a standard for other NEAsian countries to follow, particularly China. Taiwan was relatively slow off the mark in responding to industrial opportunities in the renewables sector — due in no small part to the monopoly control enjoyed by Taipower over electricity generation in the island and its long-standing support for coal-fired power and nuclear power in a regime of tight price control by the government (to minimize energy costs for Taiwanese industry).

Taiwan is an exemplary case deploying the full resources of the state working closely with the private sector in domestic and international settings to drive the creation and growth of the OWP industry. It is notable that the first step in Taiwan’s strategy for creating the OWP industry was to address the domestic electricity sector, introducing major liberalization of the sector and deregulation of the generation and distribution monopoly previously held by Taipower.

In a two-stage reform, in an initial period 2017 to 2020 Taiwan first allowed renewable energy producers to sell their power generated direct to (mainly industrial) customers. This stage complemented the strong government policies introduced to promote a solar PV industry with a target of 20 GW and a domestic OWP industry with a target of 5.5 GW by 2025. The second stage over the 6 years 2019 to 2025 is envisaged as seeing the restructuring of Taipower into a holding company with two operational entities, a power generation corporation and a transmission and distribution corporation. Both entities are designated to be owned and regulated by the Taiwan state.^20^

It has been a long-term process of reform to bring this degree of liberalization into being in Taiwan (as it has in the case of Korea with KEPCO), and it was a strategic master-stroke on the part of the Tsai Ing-wen DPP government to link it to the introduction of renewables (solar PV and OWP) as a means of driving through the reform, which can now be considered as irreversible. The state’s involvement has also been evident in the move to ensure that producers of renewable power in accordance with the government’s guidelines will not suffer economically, since if payments for green power do not reach a designated minimum the government will step in as purchaser of last resort.

The Taiwan state’s framing of the OWP industry is to create competitive conditions through liberalization, and then to set realistic targets for building the OWP farms and the domestic production chains to ensure that manufacturing operations grow in Taiwan along with the scale of the offshore wind farms.^21^ At the same time Taiwan has ensured controlled entry for international firms, like **Oersted** from Denmark, to bring capabilities that are needed to the island. Oersted currently has five projects underway in Taiwan.^22^

The development of the industry has proceeded more or less to plan. The government set an initial target of 27 GW of installed renewable capacity to be achieved by 2025, with 5.5 GW being awarded as OWP. In April 2018 the Ministry of Economic Affairs announced that seven developers had been awarded contracts calling for 738 MW of grid-connected OWP to be delivered by 2020, and with a further 3 GW to be delivered by 2025. In October 2020 at the Global Offshore Wind Summit Taiwan Virtual 2020, Taiwan’s Bureau of Energy informed that “Taiwan’s offshore wind power industry is projected to be 59 terawatt-hours annually by 2035.” The bureau estimated this development would create 57,000 jobs and attract over US$ 89.8 billion (NT$2.6 trillion) from foreign and domestic investors, resulting in a reduction of carbon emissions by 32.7 million tonnes per year.^23^

Auctions have been staged where domestic firms compete with international firms to drive down the price of delivered power; this competitive bidding process was initiated in 2018 and has been extended through the FiTs scheme. The government has already announced the next phase of development that is expected to see a further 10-GW capacity OWP developed. The critical element of this strategy is localization, with a 4-year industry promotion plan to ensure that supply chains are located within Taiwan.

### Korea

Korea is shaping up as a major player in the OWP space, particularly in light of recent government announcements. In July 2020 the Moon Jae-In administration announced a set of new targets for OWP development in Korea, setting a goal of 12 GW to be constructed by 2030, i.e., within the next decade. This is a ***100-fold expansion*** from the country’s current level of 124-MW capacity.^24^ Funds for investment are to be drawn largely from the private sector. In October 2020, The Ministry of Trade, Industry and Energy of Korea disclosed its plan to further stimulate the development of OWP projects. The measures included simplifying project approval and site selection procedures, and to improve project profitability, granting extra value to Renewable Energy Certificate for large-scale OWP projects where local governments play a leading role. Infrastructure developments are underway too. The public grid will be upgraded to connect with large-scale OWP projects and expand the capacity to 20 GW.^25^

On cue, Korea’s largest wind turbine developer, ***Doosan Heavy Industries & Construction***, announced that it was aligning its business strategy with the government’s energy transition policy. The Company is growing its offshore wind power portfolio to make it a business with annual sales of over one trillion Won (US$892 million) by 2025.^26^ The company is currently the major supplier of wind turbines in Korea, having initiated involvement with OWP since 2005, while continuing to expand its onshore wind business. Under the RE3020 Roadmap (reaching 20% renewable energy by 2030), the government set even more ambitious goals.^27^

It is notable that Korea’s national goals for OWP development are couched as business goals — direct construction targets for capacity expansion and investment targets — rather than goals in terms of carbon reductions. This setting of business targets is characteristic of the North East Asian approach — viewing the renewable energy industry promotion efforts as industry expansion plans just as much as environmental/ecological plans. This is what we mean by developmental environmentalism as characteristic of the NEAsian approach to expanding investment in clean and green energy.

### Japan

Japan has made a cautious and slow shift from its previous strategy of building a national energy system around nuclear power and fossil fuels. It was the Fukushima nuclear disaster of 11 Mar 2011 that instigated the shift. With the announcement in late 2019 of a new goal of 10 GW of offshore wind power, to be achieved by 2030,^28^ Japan has begun in earnest its clean energy transition. As a late starter it is focusing on the latest innovative wave in renewables, i.e., on the OWP sector with its near endless opportunities for strong engineering firms. The first stage of the new Japanese strategy will involve two wind farms with capacity of 140 MW, and investment of 100 billion Yen. The project sponsors are from a wide range of industries including green energies as well as fossil fuel product, together with construction, electric utility, finance, waterworks, and mechanical machine manufacture.^29^

In July 2020, METI disclosed the government’s plan to approve three to four OWP projects a year (a total of 30) in the next decade and to enhance grid operation flexibility to facilitate the renewable energy expansion. The government aims to increase the proportion of renewable power generation from 17% in 2018 to 22 to 24% by 2030.^30^ The Japanese government unveiled a new ***Offshore Wind Industry Vision*** document in late 2020 (recalling the famous “vision” documents issued by MITI in the heyday of the Japanese developmental state), where it announced plans to allocate 1 GW of OWP each year in the decade from 2020 to 2030, and an anticipated 30–45 GW of OWP cumulative capacity by 2040. The plan calls for strong development of a local supply chain (reaching 60% local supply by 2040) and cost reduction down to 8–9 Yen per kWh.^31^ It is clear that Japan intends to be a significant player in the emergent OWP industry although occurring at a slower pace than many expected (Li 2022).

### China

China is already leading its NEAsian competitors in terms of scale of its new build of OWP installations, including the first signs of strong involvement in floating platform OWP. In 2019, Chinese firms installed 24 GW^32^ of onshore wind and 2.4 GW^33^ of offshore wind capacity — representing no less than 40 percent and 39 percent of total global installations, respectively. This level of development cemented China’s position as the world’s largest market for both onshore and offshore wind, as it created new benchmarks for the other NEAsian countries to aspire to. Then in January 2020, the Chinese central government announced it would cease subsidies for offshore wind from 2022 onward, meaning that firms would have to reduce costs even faster than anticipated in order to stay competitive. Subsidies would be allowed from provincial governments which are encouraged to provide continuity for the sector. Shanghai has arranged special funds to grant cash reward to OWP project investors based on the projects’ annual electricity generation. This policy is designed to be implemented from 2020 to 2028.^34^

This is the clearest indication that the China government expects the OWP industry to be able to grow rapidly and achieve self-reliance, with a view to being able to withstand international competition. No other NEAsian power has yet to take a comparable step towards national self-reliance and international competitiveness.

In the twenty-first century China has pursued a strategy of market followership and emulation, building world-leading renewable energy industries, focused on hydro (water), wind, and sun. By 2019 China’s WWS capacities were hydro 356 GW, wind 210 GW, and solar 205 GW, making for a WWS total of 771 GW (around 39% of total generating capacity) — far in excess of other countries’ commitments.^35^

The China government pursued a determined strategy to grow the wind power industry, with an emphasis on clear targets, and local content requirements to build local supply chains. China’s first offshore wind project was launched in the first decade of the new century — Donghai Bridge project rated at 102 MW, with electricity generation coming on stream in May 2010.^36^ In the same year the government launched a tender for a first round of offshore concession projects, adding a further 1 GW of projects off the coast of Jiangsu province. By the year 2011 China had allowed installation of offshore projects rated at 242 MW, putting the country in third position after the UK (2094 MW) and Denmark (857 MW) (IRENA-GWEC, 2013). China’s progress was slowed while rights of wind power developers were negotiated with fishing and shipping maritime interests. To date, no fewer than eight Chinese turbine manufacturers have released large-scale turbines operating at more than 5-MW capacity, with six of these being listed amongst the world’s Top Ten turbine suppliers in 2019.^37^ In 2021 China’s MingYang Smart Energy released its 16-MW offshore wind power turbine, exceeding the largest exemplar produced by GE, the 14-GW Haliade.^38^ The Chinese oil major CNOOC is already a significant player in the emerging OWP industry.^39^ China announced that feed-in tariffs for OWP would expire at the end of 2021, leading to an estimated frenzy of investment in 7.5 GW of new OWP capacity.^40^

The targets set by the central government in China are complemented by ambitious targets set by provincial governments. Total targets amounting to 30 GW have been set, with Jiangsu in the lead (15 GW) followed by Zhejiang (6.5 GW), Fujian (5 GW), and Shandong (3 GW) (GWEC, 2020). Jiangsu in particular is hosting the largest offshore wind farm in China, the Rudong Offshore wind farm, being built by China Three Gorges (CTG) Corporation and which installed 3 GW of capacity in 2021.^41^ It is notable that many of these targets are associated with promotion of local supply chains. Some Chinese coastal cities promote themselves as future offshore wind manufacturing hubs — as have Yangjiang and Nantong (GWEC, 2020). Nantong has a 3-year action plan to build a complete OWP industry chain; the city expected that the industry’s revenue would exceed RMB 120 billion (US$ 18 billion) by 2022.

China is already the world’s largest manufacturing base for onshore wind, with local supply chains already in place. The industry announced commitments to reach grid parity by the end of 2020 — earlier than other established onshore wind markets. The offshore sector can be anticipated to benefit from the onshore experience in this evolutionary transition.

## The developmental-environmental drivers and dynamics of Northeast Asia’s push into OWP

Let us return to the central question posed at the outset: why it is that Japan, Korea, Taiwan, and China regard industrial strategy as something to spur the green shift and accelerate the clean energy transition? We have highlighted the D-E drivers and dynamics of NE Asia’s efforts to promote the OWP sector, and here we contrast these aspects with features that characterize the multilevel perspective (MLP) on green transitions.

### The role of government and state agencies

Our argument in this paper is that the NEAsian countries have all developed strategies for promotion of OWP as the third wave of the renewable revolution, involving state agencies and corporations in complex initiatives that are best characterized as cases of developmental environmentalism (DE). The practice of DE as exemplified by the NEAsian countries as they advance their OWP goals involve the setting of clear targets and state policies that facilitate the meeting of these targets. As of mid-2020 there are clear growth targets (official or unofficial) set by all four NEAsian countries for their emerging OWP industries (Table 1).

**Table 1:** NEAsian OWP 2025/2030 targets

Country	OWP 2025/2030 targets
Taiwan	5.5 GW by 2025
Korea	12 GW by 2030
Japan	10 GW by 2030^a^
China	No official target set yet in China GWEC 2020 predicted an addition of 52 GW by 2030 for China. The Global Offshore Wind Summit 2020 held in Jinan predicted new capacity installation of 53 GW by 2030, i.e., to reach 60 GW (compared with 7 GW in 2019)^b^

*Source*: Authors

^a^See “New laws and new targets: renewable power in Japan,” *power.nridigital*, at https://power.nridigital.com/future_power_technology_mar20/new_laws_and_new_targets_renewable_power_in_japan

^b^See “OWP in coastal provinces estimated close to 60 GW by 2030 预计到2030年, 沿海省份的海上风电目标接近60吉瓦, *Wind.IN-EN.com*, 5 September 2020, at https://wind.in-en.com/html/wind-2391366.shtml (Chinese).

What is striking is that these are all realistic business targets, set as guides for supply chain development, and financing goals — as in the creation of financing consortia as practiced by Taiwan. They are not carbon reduction targets and emission reduction achievements as such — although they will undoubtedly promote such carbon reduction goals as and when they are implemented. This focus on business goals as central state development target is characteristic of East Asian strategic industrial goals.

### The economics of OWP: hybridized industrial ecosystems

The significance of these national business targets is that they map out a pathway for new industry creation in the evolving political economy of NEAsia. It is not the state agencies that will create the new industry but firms through repeated processes of manufacturing learning curves — some of which are SOEs but most of which are private firms — working with the flow of market forces. Our way of capturing this pattern of accelerated learning is to describe it as being guided by the deliberate creation of hybridized industrial ecosystems (HIEs) (Kim 2019). These are evident in the promotion of local supply chains feeding into the emergent OWP sector in the NEAsian countries, spanning activities such as wind turbine production and components, platform building, and the adaptation of supply vessels from the needs of oil and gas drilling operations to wind power generation operations.

### Sociotechnical transitions: developmental environmentalism

Two decades ago the Dutch-UK scholar Prof Frank Geels launched a new trend in analyzing sociotechnical transitions with his analysis of them as involving interactions between three levels of analysis, namely (1) emerging niche markets, (2) the incumbent sociotechnical regime, and (3) the prevailing sociotechnical landscape (Geels 2002; Geels et al. 2017). This has proven to be a fruitful framework for analysis, enlivened by telling case studies such as the shift in the UK in the nineteenth century from sailing ships to steamships, or the shift in The Netherlands from communal cesspools to sewerage systems driven by public hygiene concerns. While recognizing the utility of this MLP framework, we find that it has little insight when it comes to East Asian sociotechnical transitions where state agencies play a central role — perhaps because of its emphasis on the micro-dynamics of innovation and transitions and less emphasis on the role of driving actors like states. In Geels’ founding case study of the shift from sailing ships to steamships, the central role played by the British Navy is mentioned only in passing, whereas it was the Navy that defined the scope of the transition by its navigational rules and standards.^42^ Our argument in this paper is that state agencies are playing a similar role in NEAsia by their setting rules and standards that both domestic and foreign companies must adhere to, and where state agencies for R&D, for procurement, for public auctions, and for skill development, all play their role in shaping the transition.

### Fossil fuel companies’ diversification strategies: new wine in old bottles?

The interesting feature of the strategies being deployed in the OWP sector is the role played by oil and gas companies building on their existing capabilities and infrastructure (e.g., floating drilling platforms) in order to pursue diversification strategies and move to become renewable power companies. While these tendencies are not yet clearly evident in the NEAsian countries they are certainly evident in Europe, where companies like Equinor from Norway, Total from France, and BP are already moving to deploy their floating platform technology as foundation for their shift across to building OWP businesses. The Norwegian state-owned company Equinor (formerly Statoil) is a leader in this transition.

The NEAsian competitors can be expected to deploy their full arsenal of strategies to emulate and adapt these initiatives as they become available, e.g., through joint ventures. In NEAsia there are already signs pointing in the direction of this kind of diversification. The Korean National Oil Co. (KNOC) has already embarked on a floating platform OWP project in its existing Donghae-1 gas field, as noted above. The Chinese National Offshore Oil Corporation (CNOOC) has announced plans to enter the OWP, such as participation in a 300-MW project off the coast of Jiangsu Province.^43^ Singapore’s Keppel Corporation is also moving into the space, with its securing contracts to build supply vessels and electrical transformers for OWP projects.^44^

In this way the incumbent oil and gas companies and engineering companies entering the OWP sector are validating studies in the business literature on diversification and the role of resource access (Nelson and Winter 1982; Hill and Rothaermel 2003) as the incumbents redeploy their resources and capabilities.

### Creative destruction in the offshore wind power industry

In 1942 the acclaimed political economist Joseph Schumpeter published his classic study, *Capitalism, Socialism and Democracy*, containing a short chapter 7 “The process of creative destruction.” Schumpeter argued, drawing on contemporary and previous industrial episodes, that as new industries are fashioned they create new profit opportunities that displace the profits earned by incumbents.^45^ The very same process of creative destruction may be observed in the global green shift that is underway.^46^ The OWP sector adds its own complication to this process by providing an explanation for the involvement of oil and gas companies in this transition, strategically redeploying their fossil fuel capabilities in the new setting of renewables. This case illustrates the proposition that creative destruction does not have to mean that all incumbents are destroyed. On the contrary there are opportunities generated for incumbents by the process that enable them to utilize their existing skills, technologies, and capabilities in the new setting of renewable power.

## Concluding remarks

As debate over the next wave of renewable power heats up, the claims of offshore wind power are becoming increasingly important. The scale of the devices needed for OWP is greater than for onshore solar and wind power; indeed, the emergent OWP industry resembles shipbuilding and heavy engineering (roads, bridges, tunnels) more than it does mass production industries. In this setting it is clear that NEAsian countries, led by China’s impressive performance, but also Taiwan, Korea, and Japan, in making substantive progress, look set to overtake the early leaders from Europe as they produce larger and larger turbines, and bigger and bigger platforms. These trends can be expected to accelerate as the NEAsians seek to extend to the open seas with floating wind platforms.

The argument of this paper is that OWP is shaping up to become an arena of great power competitive rivalry, as leading firms from NEAsia, the EU, and the USA engage in strategic competition to secure and maintain a share of this evolving political economy as it emerges over the course of the next decade. The competitive dynamics will turn on the firms and countries with the best technology, certainly, and the smartest entrepreneurs, but also on those with the smartest and most resolute industrial strategies, shaping markets for the future and honing tax and policy incentives in ways designed to grow the industry at maximal sustainable rate. We have sought to demonstrate that OWP is the setting where these state-based strategies of developmental environmentalism emerging in NEAsia are likely to prove to be most fruitful and productive.

Our central point is that while each of the NEAsian countries is drawing on prior state-led strategies for industrial transformation as they enter the new OWP era, this does not imply that they necessarily pursue uniform strategies. The differences between the NEAsian countries’ approaches to OWP are as arresting as their commonalities in the application of developmental environmental strategies.

In the terms of our argument OWP is doubly important. In the first place it is likely to be the next wave of renewables, promising to become one of the largest renewables sectors of all. But even more importantly it is likely to be the sector where oil and gas companies can be expected to become major players, bringing a new depth to the competitive rivalry anticipated in this emergent sector. This puts the Schumpeterian creative destruction that accompanies new industry emergence in a fresh light, revealing how the oil and gas companies, with their vast experience of building offshore drilling platforms and the supply chains that service them, are being presented with the opportunity to diversify from oil and gas operations to renewable power operations and ultimately to green hydrogen operations.

This is the long-awaited opportunity for the oil and gas majors, and we have pointed to the emergence of early movers in this diversification strategy in the form of companies like Equinor from Norway and Total from France, being joined by others such as BP, and with CNOOC, KNOC, and Mitsubishi entering the sector from NEAsia. A new era in great power rivalry is clearly emerging, characterized by direct involvement of the oil and gas industries — with their enormous resources and political influence — in the clean and green transition, and where NEAsian firms and governments promise to be central players.

## Footnotes

1 See *Global Offshore Wind Report 2020*, Global Wind Energy Council, Aug 2020, at https://gwec.net/global-offshore-wind-report-2020/

2 See *Global Offshore Wind Report 2021*, Global Wind Energy Council, May 2021, at https://gwec.net/global-wind-report-2021/

3 See, e.g., “Floating wind farms — the power source of the future,” *The Guardian*, Sat 8 August 2020, at https://www.theguardian.com/news/2020/aug/07/weatherwatch-floating-wind-farms-the-power-source-of-the-future. For scholarly treatment of the OWP, see, for example, MacKinnon et al. (2019). For comparative analyses of sustainability transitions in China, Japan, and California, see Trencher et al. (2021).

4 In the year 2017 China added 1.2 GW in OWP while Europe added 3.2 GW. The next year, 2018, China added 1.6 GW while Europe added 2.7, and in 2019 China added 2.4 GW (i.e., 40%) while Europe added 3.6 GW (60%), with just 0.1 GW added by others — to make the record 6.1 GW added for that year.

5 See “World installs 6.1 GW of offshore wind in 2020, led by China,” by GWEC, Sep 2021, at https://gwec.net/world-installs-6-1gw-of-offshore-wind-in-2020-led-by-china/

6 The situation in 2025 is anticipated to be annual capacity additions as follows: Europe 8.7 GW, China 5.0 GW, North America 4.2 GW, and Asia (for example, China) 3.7 GW.

7 See “Wind Energy Beijing Declaration’ releases positive policy signals after the installation trend, equipment manufacturers are still optimistic about the expansion of production capacity” *SMM News*, 15 October 2020, at https://news.metal.com/newscontent/101289066/%22wind-energy-beijing-declaration%22-releases-positive-policy-signals-after-the-installation-trend-equipment-manufacturers-are-still-optimistic-about-the-expansion-of-production-capacity

8 See “Launch of New Hydrogen Utilization Council in Kobe/Kansai area,” *Marubeni*, 4 September, 2020, at https://www.marubeni.com/en/news/2020/release/00010.html#:~:text=Kobe%2FKansai%20area%2C%20Japan%20%E2%80%93,and%20establishing%20a%20hydrogen%20supply as well as. “Industry seeks to replicate Taiwan wind success throughout Asia,” 30 October 2020, *Petroleum Economist*, at https://www.petroleum-economist.com/articles/low-carbon-energy/energy-transition/2020/industry-seeks-to-replicate-taiwan-wind-success-throughout-asia

9 See “Falling cost of green hydrogen boosts South Korea’s clean energy agenda,” *NS Energy*, 24 August 2020, at https://www.nsenergybusiness.com/features/south-korea-green-hydrogen/

10 See New offshore wind-powered hydrogen project approved in Germany,” by Joshua Hill, *RenewEconomy*, 5 August 2020, at. New offshore wind-powered hydrogen project approved in Germany | RenewEconomy.

11 See “Floating wind turbines on the rise: NREL expert discusses future powered by floating offshore wind,” National Renewable Energy Laboratory, 2 April 2020, at https://www.nrel.gov/news/program/2020/floating-offshore-wind-rises.html and “U.S. offshore wind pipeline tops 24 GW,” *Offshorewind.biz*, August 9 2017, at https://www.offshorewind.biz/2017/08/09/u-s-offshore-wind-pipeline-tops-24gw/

12 See “France oil giant total enters floating offshore wind sector with UK acquisition,” *RenewEconomy*, 24 March 2020, at https://reneweconomy.com.au/france-oil-giant-total-enters-floating-offshore-wind-sector-with-uk-acquisition-35866/

13 See “BP enters offshore wind with Equinor deal,” *Financial Times*, Sep 10 2020, at https://www.ft.com/content/51507855-183d-4840-a2e6-4ba3b12941cb

14 See “China’s CNOOC launches first offshore wind plant,” *Reuters*, Sep 15 2020, at China’s CNOOC launches first offshore wind power plant | Reuters.

15 See “South Korean firms turn to offshore wind power,” REVE, 21 Sep 2020, at https://www.evwind.es/2020/09/21/south-korean-firms-turn-to-offshore-wind-power/77266

16 But Japan has not eclipsed China in terms of installed offshore wind power installations, as argued by Li (2022).

17 See “Japan industry and policy news: offshore wind technology,” EU-Japan Centre, at https://www.eu-japan.eu/news/japan-industry-and-policy-news-offshore-wind-technology

18 See “Mitsubishi Heavy to make offshore wind turbines in the US and Asia,” *Nikkei Weekly*, 12 July 2019, at https://asia.nikkei.com/Business/Companies/Mitsubishi-Heavy-to-make-offshore-wind-turbines-in-US-and-Asia

19 See https://www.reutersevents.com/renewables/wind-energy-update/floating-wind-built-series-could-be-cheaper-fixed-bottom

20 See *The Law Review* “Spotlight: the energy markets in Taiwan,” Aug 6 2020, at https://thelawreviews.co.uk/edition/the-energy-regulation-and-markets-review-edition-9/1228315/taiwan

21 On the role of the state as developmental agent in Taiwan’s creation of an OWP industry, see Chien (2019).

22 The projects include Formosa 1, stages 1 and 2, and the Changhua project. See Oersted, at https://orsted.tw/en/orsted-in-taiwan/our-projects

23 See “Wind industry seeks more governmental flexibility,” *Taipei Times*, 13 October 2020, at https://www.taipeitimes.com/News/biz/archives/2020/10/13/2003745053

24 See “Moon vows bold investment in offshore wind power industry,” *Korea Herald,* 17 July 2020, at http://www.koreaherald.com/view.php?ud=20200717000624

25 See “The South Korean government began to reform site selection and green certificate subsidy systems for offshore wind power projects,” *Wind.IN-EN.com*, 3 November 2020, at https://wind.in-en.com/html/wind-2394352.shtml (Chinese).

26 See “Doosan Heavy to join hands with KNOC for offshore wind farms,” *Korea Herald*, 10 Sep 2020, at http://www.koreaherald.com/view.php?ud=20200910000814 and “Charting the latest offshore wind developments in South Korea,” *NS Energy*, 9 November 2020, at https://www.nsenergybusiness.com/news/south-korea-offshore-wind/

27 In the RE3020 roadmap the government set the goal of adding 48.7 GW of newly installed capacity to the country’s existing 15.1 GW (as of 2017) by 2030 for a combined figure of 63.8 GW of green power. See Kim (2020), p. 6.

28 See “Japan aims for 10 GW offshore wind energy capacity by 2030,” *reve*, 30 July 2020, at https://www.evwind.es/2020/07/30/japan-aims-for-10-gw-offshore-wind-energy-capacity-by-2030/76153#:~:text=July%2030%2C%202020%20reve,30%2D45%20GW%20in%202040.

29 Marubeni, Obayashi, Tohoku Sustainable & Renewable Energy, Cosmo Eco Power, The Kansai Electric Power, Chubu Electric Power, The Akita Bank, Ohmori, Sawakigumi, Kyowa Oil, Katokensetsu, Kanpu, and Sankyo are on the list. See “Special report: offshore wind power project in Japan,” Mori, Hamada & Matsumoto, 6 Mar 2020, at https://www.mhmjapan.com/en/legal-topics/year/2020/1.html

30 See “Japan seeks massive jump in offshore wind power over 10 years,” Nikkei Asia, 9 July 2020, at https://asia.nikkei.com/Business/Energy/Japan-seeks-massive-jump-in-offshore-wind-power-over-10-years2#:~:text=TOKYO%20%2D%2D%20The%20Japanese%20government,decade%20beginning%20next%20fiscal%20year

31 See “Japan plans 45 GW offshore wind power by 2040,” *Pinsent Masons*, at https://www.pinsentmasons.com/out-law/news/japan-plans-45gw-offshore-wind-power-by-2040

32 See GWEC, 5 July 2020, at https://www.evwind.es/2020/07/05/wind-energy-expanded-19-in-2019-with-around-60-gw-of-new-capacity/75563

33 See *Global Offshore Wind Report 2020*, Global Wind Energy Council, Aug 2020, at https://gwec.net/global-offshore-wind-report-2020/

34 See “Measures of Shanghai to support the development of renewable energy and new energy with special funds (2020 edition), Shanghai Municipal Development and Reform Commission, 8 June 2020, at http://fgw.sh.gov.cn/zgjjl/20200608/e186fb40e413471eb32e3783c207f0f1.html (accessed on 22 Sep 2020).

35 See John Mathews and Carol X. Huang, Greening trends within China’s energy system: a 2019 update, *Asia Pacific Journal: Japan Focus*, Sep 2020, at https://apjjf.org/2020/17/MathewsHuang.html

36 See Project profile: Donghai bridge project, *Renewable Energy World*, March 2011, at https://www.renewableenergyworld.com/2011/03/23/project-profile-shanghai-donghai-bridge/#gref

37 See https://gwec.net/wind-turbine-sizes-keep-growing-as-industry-consolidation-continues/

38 See https://electrek.co/2022/02/22/a-chinese-company-is-building-a-colossal-16-mw-offshore-wind-turbine/

39 See “China’s CNOOC launches first offshore wind power plant,” *Reuters*, 15 September 2020, at https://www.reuters.com/article/china-cnooc-idUSL4N2GC2W8

40 See “World installs 6.1 GW of offshore wind in 2020, led by China,” *GWEC*, 9 Sep 2021, at World installs 6.1 GW of offshore wind in 2020, led by China — Global Wind Energy Council (gwec.net).

41 See “China’s largest offshore wind farm helps propel its green targets,” by Liu Wei, *CGTN*, 29 Oct 2021, at China’s largest offshore wind farm helps propel its green targets — CGTN. See also the media release by the Rudong Government “Largest offshore wind farm in Asia built in Rudong” at http://www.rudong.gov.cn/rdxrmzf/rdyw/content/c1f817f3-8b77-4238-aea5-e91ee4d2476e.html (in Chinese).

42 For the original contribution by Geels that launched the MLP, see Geels (2002). For the mature enunciation of the MLP, see Geels and Schot (2007). For a discussion of its application to the green transition, see Geels, Sovacool et al. (2017). For a debate over criticized aspects of the MLP (but not engaging with our critique of its absence of state agencies in NEAsia), see Geels (2011).

43 We mention the cases of KNOC and CNOOC to illustrate how incumbents can diversify their operations under state guidance to take advantage of fresh opportunities such as those created in the OWP sector. See “CNOOC opens first offshore wind power farm with 300 MW capacity,” NASDAQ, 17 Sep 2020, at https://au.sports.yahoo.com/cnooc-opens-first-offshore-wind-130801622.html

44 See “Keppel O&M unit bags $560 m offshore wind farm deal,” *Straits Times*, 9 May 2019, at https://www.straitstimes.com/business/keppel-om-unit-bags-560m-offshore-wind-farm-deal

45 See Schumpeter 1942 (1950), Chapter 7, pp. 81–86.

46 The shift involved may be characterized as supersession of one set of industrial platforms by another, as argued by Mathews (2020).

## References

[CR1] Bettin SS (2020) Electricity infrastructure and innovation in the next phase of energy transition—amendments to the technology innovation system framework. Rev Evol Polit Econ 1:371–395

[CR2] Chien K-H (2019) Pacing for renewable energy development: the developmental state in Taiwan’s offshore wind power, Ann Am Assoc Geogr 110 (3), at https://www.tandfonline.com/doi/abs/10.1080/24694452.2019.1630246?journalCode=raag21

[CR3] DeCastro M, Salvador S, Gomez-Gesteira M, Costoya X, Carvalho D, Sanz-Larruga FJ, Gimeno L (2019) Europe, China and the United States: three different approaches to the development of offshore wind energy. Renew Sustain Energy Rev 109:55–70 10.1111/nyas.13924 30008177

[CR4] Geels FW (2002) Technological transitions as evolutionary reconfiguration processes: a multi-level perspective and a case study. Res Policy 31:1257–1274

[CR5] Geels FW (2011) The multi-level perspective on sustainability transitions: responses to seven criticisms. Environ Innov Soc Trans 1:24–40

[CR6] Geels FW, Schot J (2007) Typology of sociotechnical transition pathways. Res Policy 36:399–417

[CR7] Geels FW, Sovacool BK, Schwanen T, Sorrell S (2017) Sociotechnical transitions for deep decarbonization. Science 357(6357):1242–1244 28935795 10.1126/science.aao3760

[CR8] GWEC (2020) Global Offshore Wind Report 2020. Available at https://gwec.net/global-offshore-wind-report-2020/

[CR9] Hill CW, Rothaermel FT (2003) The performance of incumbent firms in the face of radical technological innovation. Acad Manag Rev 28(2):257–274

[CR10] IRENA-GWEC (2013) 30 years of policies for wind energy: lessons from China*.* Available at https://www.irena.org/-/media/Files/IRENA/Agency/Publication/2013/GWEC/GWEC_China.pdf?la=en&hash=E1FC001617FB7AB786A7D831D271853B9404449A (accessed 1 Nov. 2020)

[CR11] Kim S (2019) Hybridized industrial ecosystems and the makings of a new developmental infrastructure in East Asia’s green energy sector. Review of International Political Economy 26(1):158–182

[CR12] Kim SY, Thurbon E (2015) Developmental environmentalism: explaining South Korea’s ambitious pursuit of green growth. Polit Soc 43(2):213–240

[CR13] Kim S (2020) National competitive advantage and energy transitions in Korea and Taiwan, New Polit Econ at 10.1080/13563467.2020.1755245

[CR14] Kuzemko C, Lawrence A, Watson M (2019) New directions in the international political economy of energy. Review of International Political Economy 26(1):1–24

[CR15] Li A (2022) Centralization or decentralization: divergent paths of governing offshore wind between China and Japan. Energy Res Soc Sci 84:102426

[CR16] MacKinnon D, Dawley S, Steen M, Menzel MP, Karlsen A, Sommer P, Hansen GH, Normann HE (2019) Path creation, global production networks and regional development: a comparative international analysis of the offshore wind sector. Prog Plan 130:1–32

[CR17] Mathews JA (2020) Schumpeterian economic dynamics of greening: propagation of green eco-platforms. J Evol Econ 30:929–948

[CR18] Mathews JA, Tan H (2014) Economics: manufacture renewables to build energy security. Nature 513(7517):166–168 25209783 10.1038/513166a

[CR19] Nelson RR, Winter SG (1982) An evolutionary theory of economic change. Harvard University Press, Cambridge, MA

[CR20] Newell P (2019) *Trasformismo* or transformation? The global political economy of energy transitions. Rev Int Polit Econ 26(1):25–48

[CR21] Pearse R (2020) Theorising the political economy of energy transformations: agency, structure, space, process. New Political Economy 26(6):951–963

[CR22] Schumpeter JA (1942/1976) Capitalism, socialism and democracy London: Routledge

[CR23] Thurbon E, Kim SY, Mathews JA, Tan H (2021) More ‘creative’ than ‘destructive’? Synthesizing Schumpeterian and developmental state perspectives to explain mixed results in Korea’s clean energy shift. Journal of Environment and Development. 10.1177/10704965211013491

[CR24] Trencher G, Truong N, Temocin P, Duygan M (2021) Top-down sustainability transitions in action: how do incumbent actors drive electric mobility diffusion in China, Japan, and California? Energy Res Soc Sci 79:102184

